# Effect of Peripheral Electrical Stimulation (PES) on Nocturnal Blood Glucose in Type 2 Diabetes: A Randomized Crossover Pilot Study

**DOI:** 10.1371/journal.pone.0168805

**Published:** 2016-12-20

**Authors:** Merav Catalogna, Keren Doenyas-Barak, Roi Sagi, Ramzia Abu-Hamad, Uri Nevo, Eshel Ben-Jacob, Shai Efrati

**Affiliations:** 1 Department of Biomedical Engineering, Faculty of Engineering, Tel Aviv University, Tel Aviv, Israel; 2 Research and Development Unit, Assaf Harofeh Medical Center, Zerifin, Israel, affiliated with the Sackler Faculty of Medicine, Tel Aviv University, Tel Aviv, Israel; 3 School of Physics and Astronomy, Raymond & Beverly Sackler Faculty of Exact Sciences, Tel Aviv University, Tel Aviv, Israel; Garvan Institute of Medical Research, AUSTRALIA

## Abstract

**Background:**

Regulation of hepatic glucose production has been a target for antidiabetic drug development, due to its major contribution to glucose homeostasis. Previous pre-clinical study demonstrated that peripheral electrical stimulation (PES) may stimulate glucose utilization and improve hepatic insulin sensitivity. The aim of the present study was to evaluate safety, tolerability, and the glucose-lowering effect of this approach in patients with type 2 diabetes (T2DM).

**Methods:**

Twelve patients with T2DM were recruited for an open label, interventional, randomized trial. Eleven patients underwent, in a crossover design, an active, and a no-intervention control periods, separated with a two-week washout phase. During the active period, the patients received a daily lower extremity PES treatment (1.33Hz/16Hz burst mode), for 14 days. Study endpoints included changes in glucose levels, number of hypoglycemic episodes, and other potential side effects. Endpoints were analyzed based on continuous glucose meter readings, and laboratory evaluation.

**Results:**

We found that during the active period, the most significant effect was on nocturnal glucose control (*P* < 0.0004), as well as on pre-meal mean glucose levels (*P* < 0.02). The mean daily glucose levels were also decreased although it did not reach clinical significance (*P* = 0.07). A reduction in serum cortisol (*P* < 0.01) but not in insulin was also detected after 2 weeks of treatment. No adverse events were recorded.

**Conclusions:**

These results indicate that repeated PES treatment, even for a very short duration, can improve blood glucose control, possibly by suppressing hepatic glucose production. This effect may be mediated via hypothalamic-pituitary-adrenal axis modulation.

**Trial registration:**

ClinicalTrials.gov NCT02727790

## Introduction

Type 2 diabetes mellitus (T2DM) is a metabolic disease characterized by chronic hyperglycemia. The key pathophysiologic abnormalities that have been associated with the disease are decreased peripheral glucose utilization combined with augmented endogenous glucose production [1, 2]. In T2DM patients, fasting hyperglycemia is strongly correlated with increased whole body glucose production, and is mainly attributed to an increased rate of gluconeogenesis [1, 3, 4]. The contribution of increased fasting glucose levels to the overall diurnal hyperglycemia increases gradually with diabetes progression [5]. Several cellular signaling and transcriptional pathways are involved in the regulation of hepatic gluconeogenesis. Gene expression of gluconeogenic enzymes, such as phosphoenolpyruvate carboxykinase (PEPCK) and glucose-6-phosphatase (G6Pase), is hormonally upregulated by glucagon and by glucocorticoids, and is downregulated by insulin under fed conditions [6]. In addition to this direct hepatic action, central-mediated, neuroendocrine, mechanisms are also involved in the regulation of gluconeogenesis. Three neuroendocrine pathways between the hypothalamus and the liver are known to be related to gluconeogenesis. (i) Hypothalamic-hepatic axis–Gluconeogenesis is being affected through direct hepatic innervation by both the sympathetic system, through the splanchnic nerve, and the parasympathetic system, through the vagal nerve [7, 8]. These pathways are stimulated by the ventromedial and the lateral hypothalamic areas respectively. (ii) Hypothalamic-pancreatic axis–The hypothalamus modulates the insulin-glucagon ratio through a direct pancreatic innervation [9]. In addition to the direct action of these pancreatic hormones on hepatocytes, an indirect effect also exists. As for example, hypothalamic insulin sensing is essential for inhibition of gluconeogenesis [10]. (iii) Hypothalamic-pituitary-adrenal axis–The hypothalamus regulates both the circadian pattern, and the absolute levels of adrenocorticotropic hormone (ACTH) secretion which in turn stimulates the release of glucocorticoid hormones [11].

Electro-therapy is widely used in clinical practice for the treatment of a variety of medical disorders including cardiac diseases, spinal cord and peripheral nerve disorders, and pain. [12–14]. Lately, there is a growing evidence for the beneficial effect of peripheral electro-therapy on glycemic control. The efficacy of repeated electro-acupuncture (EA) treatment for reducing baseline glucose levels, and improving insulin sensitivity has been well studied and demonstrated in animal models [15–18]. EA applies a low frequency (1–25 Hz) stimulation through needle electrodes inserted in traditional skin acupuncture points for 30–90 min per treatment. Nevertheless, only few studies examined the effect of such treatment in humans. Repeated EA treatments have been shown to reduce body weight, and increase serum insulin, and c-peptide levels in obese women [19]. Five weeks of EA treatments have been recently shown to improve HbA1c levels, circulating and adipose tissue androgens in women with polycystic ovary syndrome [20]. A decrease in fasting glucose levels in obese women [21] and increased glucose disposal rate in normal subjects [22] were observed in response to a single treatment. However, no significant change in fasting glucose levels was found in women with polycystic ovary syndrome [23], and in T2DM patients when applied in combination with Rosiglitazone [24], possibly due to a low number and frequencies of treatments.

A recent clinical study examined the effect of transcutaneous electrical nerve stimulation (TENS), applied noninvasively at the lower extremity through adhesive electrodes, on glycemic control during general anesthesia [25]. The study indicated that 30 min TENS might ameliorate the expected increase in blood glucose during anesthesia. Additionally, Catalogna et al. have recently shown, that noninvasive peripheral electrical stimulation (PES) treatment of a very short duration (2–3 min) may stimulate glucose utilization and improve hepatic insulin sensitivity in rats [26]. Taken together, glycemic control pathways, triggered by PES, may be related to endogenous glucose production.

In this study, we continue the work of Catalogna et al. [26], and assess the safety and tolerability of daily PES treatment performed by the patients at home. We also aimed to evaluate the effect of the treatment on glucose production and utilization as an additional potential treatment strategy for T2DM.

## Methods

### Patients

Patient eligibility criteria included a diagnosis of T2DM [2] at least 12 months prior to enrollment, Body Mass Index BMI ≤ 35 kg/m^2^, and a stable diabetes treatment regimen for at least one month prior to randomization. Patients were required to stay on the same regimen during the study. Exclusion criteria included evidence of liver, renal, or cardiovascular disease during the last 6 month before the enrollment, a permanent pacemaker, and treatment with steroids or beta-blockers. Written informed consent was obtained from all patients prior to enrollment. Then, the patients were randomly assigned to one of the two study groups according to a computer-generated randomization list. The study was approved by the Institutional Medical Ethics Committee (No. 129/12) and by the Israeli Ministry of Health Ethics Committee (HTA6446), on April 2013. This study is registered with ClinicalTrials.gov, number NCT02727790. Due to a technical misunderstanding, there was a delay in registering this study after enrolment of participants started. The authors confirm that all ongoing and related trials for this intervention are registered. Follow-up was completed in June 2016.

### Study Design

An interventional, open label, randomized, crossover trial was conducted at the Research Unit of Assaf Harofeh Medical Center. Each subject underwent an active treatment period, and a control trial period, using a randomized crossover design, with a two-week washout period separating each trial. During the active period, the patients received daily PES treatment at home for 14 days (detailed description of the PES follows). Interstitial glucose was monitored throughout the 4 weeks of the study using a FreeStyle Navigator (Abbott Diabetes Care, Alameda, CA) continuous glucose monitoring (CGM) system, for 5 days a week. Measurements with the FreeStyle Navigator system were found to be consistent, and accurate, compared with venous measurements made using a laboratory reference method, over the 5 days of sensor wear [27]. In addition, for ease of the calibration procedure, and to avoid user errors, this system has a built-in blood glucose (BG) Meter. Patients were educated on a proper use and calibration of the CGM, as instructed by the manufacturer.

**Fig 1:**
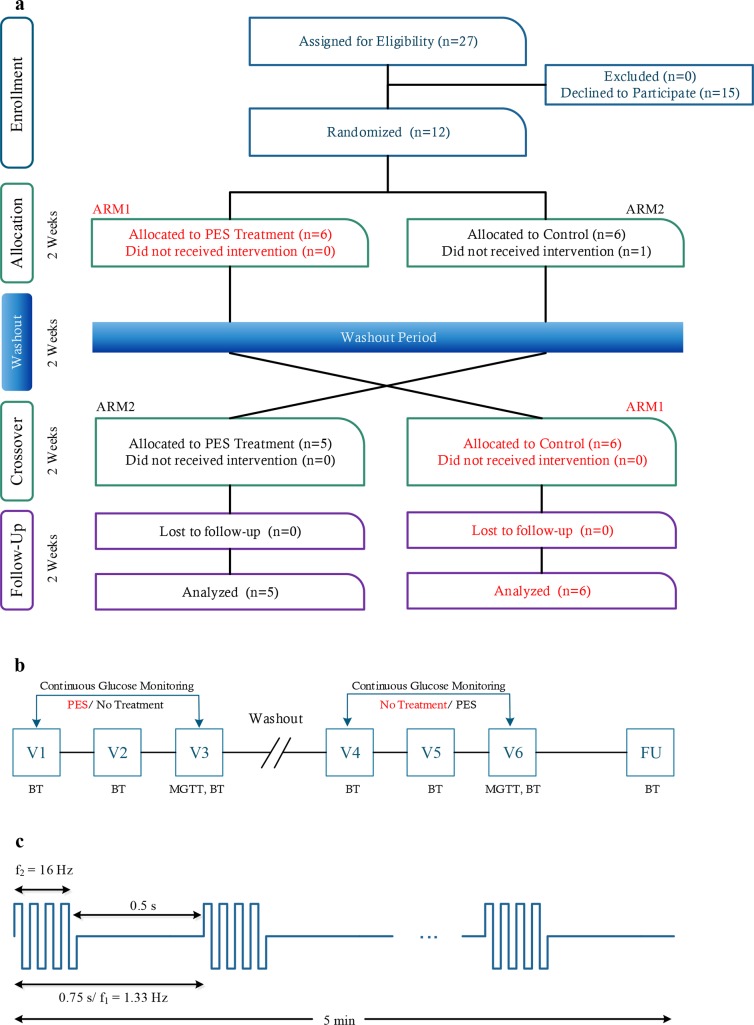
CONSORT flow chart and study design. a. A Crossover design with a two-week washout period separating each trial. b. Summary of study visits and procedures. PES–Home care peripheral electrical stimulation treatment, MGTT–meal glucose tolerance test. In visits V1-V6 and the follow up (FU) visit, all patients underwent a clinical evaluation, and BT (blood tests) were taken. c. PES signal treatment pattern.

During a weekly visit, patients underwent comprehensive clinical and laboratory evaluation: physical examination, body weight measurements, ECG, and blood pressure measurements. Laboratory tests were performed after 8 h overnight fasting, between 7:00 to 8:00 AM. Blood samples were collected for glucose, HbA1c, insulin, cortisol, and glucagon, lipid profile, and liver and renal function (ALT-Alanine aminotransferase, and AST-Aspartate aminotransferase, Bilirubin, Albumin, Creatinine, and Urea). The area of skin under the electrodes was assessed and photographed, and the CGM data was downloaded. At the end of each period (Treatment/ Control), insulin sensitivity was evaluated by using the meal glucose tolerance test (MGTT). After breakfast (Ensure Plus, Abbott 355 calories/50 g carbohydrate) was provided, blood glucose levels were monitored prior to breakfast and then every 30 min for 2 h. The primary endpoint of the study was the number of hypoglycemic episodes, and other adverse events that can be related to treatment. Secondary endpoints included minor side effects, such as changes in skin conditions under the electrode, and uncomfortable sensation during the PES treatment, and changes in blood glucose levels. A general schematic diagram of the study design is illustrated in Fig 1.

### Home Care Treatment and Glucose Monitoring

Two pre-gelled self-adhesive electrodes (*d* = 25 mm) were placed bilaterally on the anterior aspect of the legs, below the knee cap, and lateral to the anterior crest of the tibia (corresponding to Zusanli acupuncture point). This location is in proximity to the common peroneal and tibial branches of the sciatic nerve [28]. The treatment protocol was based on prior knowledge of the related Catalogna et al. animal model studies [26], and the available safety information related to the use of the device for other indications. Treatment points were located and marked by a trained study member during the first treatment visit. Electrical current was applied for 5 min (Device: Intellistim, BE-28T, Italy, Frequency 1.33Hz/ burst mode of 16Hz rectangular bi-phasic, pulse width: 150μs, see Fig 1) every morning before breakfast, during the active period of the study (2 weeks). The positive wire was connected to the right electrode and the negative was connected to the left electrode. The applied current within the electrical stimulation was adjusted individually, to evoke a weak sensation beneath the electrodes, without pain or discomfort and without muscle contraction (5–10 mA variable). Patients were asked to fill out a food diary throughout the study periods. In addition, patients were asked to record information about intense physical activity, stressful event, illness, missed medications, etc.

Assessment of glycemic control in T2DM patients is currently provided by three measures: HbA1c, an integration of glucose levels over 3 months period, fasting glucose level, and postprandial glucose levels [29]. However, these measures fail to provide comprehensive information about inter- and intra-day variations, about episodes of hypoglycemia or hyperglycemia, nor about the rate of change in glucose levels in response to external (food intake, exercise) or internal (liver function, emotional) stimuli. The use of continuous glucose monitoring (CGM) offers better interpretation of glucose dynamics through signal processing analysis.

We extracted two types of measures from the CGM time series. (a) Statistical measures and temporal events: mean BG over different time intervals (24 h, before and after meals, day, night), the area under the curve for glucose levels > 180 mg/dl, maximum and minimum levels [30]. (b) Glucose variability and risk assessment: SD (extensively used), M-value (weighted SD) Mean amplitude of glycemic excursion (MAGE), Mean of daily differences (MODD) and CONGAn (SD of the difference between values obtained exactly n hours apart) [31, 32]. Full details and equations are presented in S1 Table. The CGM analysis was performed using Matlab® v.8.2 (The Mathworks, Natick, MA).

### Assays

Plasma samples were divided into aliquots and stored at −80°C until assayed. Serum insulin was analyzed, in patients not treated with insulin, with a human insulin ELISA kit (RayBiotech, Norcross, GA, USA). Serum cortisol was analyzed using the parameter cortisol immunoassay ELISA kit (R&D Systems Inc. Minneapolis, USA). Serum glucagon was analyzed with Human Glucagon ELISA kit (RayBiotech, Norcross, GA, USA). Lipid profile, liver and renal function were measured by routine laboratory methods.

### Statistical Analysis

This pilot study was designed for assessment of feasibility criteria, and for pilot estimation of the effect needed for size calculation for a later clinical trial. We followed the recommendations of Julious [33] and Hertzog [34] for a sample size of 12 patients for a crossover design. Endpoints data were evaluated by using the crossover analysis of variance as described by Jones and Kenward [35]. No evidence of carryover or period effects were found. Comparisons between treatment and control periods were performed using the paired nonparametric Wilcoxon signed rank test or the paired student's t-test with two-tail distribution when a normality assumption holds according to Kolmogorov-Smirnov test. Unless otherwise stated, data are given as mean ± SEM. A value of *P* ≤ 0.05 is considered significant. Statistical analysis was performed with the Matlab® v. 8.2 Statistics Toolbox.

## Results

### Subject clinical and biochemical characteristics

Overall, 27 eligible patients with T2DM were identified for this study during routine clinical visits at the Assaf Harofeh Medical Center, between June 2014 and February 2015. Fifteen patients declined to participate prior to allocation after understanding the study protocol requirements. Twelve T2DM patients were included (age 45–75 years old, average 62 ± 11). One patient was dropped out due to a back injury. Three of the patients were treated with insulin and the remaining were treated with oral medications (eight patients were taking metformin and three of them in combination with sitagliptin). Baseline clinical characteristics, hormone, and lipid profile of the patients are shown in Table 1.

**Table 1:** Baseline patients' characteristics.

n (M/F)	12 (10/2)
Age (years)	62 ± 11
Duration of diabetes* (years)	12.6 ± 10.4
Weight (kg)	89.4 ± 15.0
Body-mass index (kg/m^2^)	30.5 ± 4.3
Systolic blood pressure (mm Hg)	133 ± 14.4
Diastolic blood pressure (mm Hg)	80.0 ± 9.2
HbA_1C_-NGSP %	7.3 ± 0.7
HbA_1C_-IFCC (mmol/mol)	55.9 ± 8.2
Fasting blood glucose—FBG (mg/dl)	151.5 ± 12.6
Insulin (μIU/ml)	4.6 ± 0.7
Cortisol (ng/ml)	169.4 ± 16.9
HDL-cholesterol (mg/dl)	41.5 ± 10.7
LDL-cholesterol (mg/dl)	88.3 ± 28.9
Triglycerides (mg/dl)	142.6 ± 50.9
Urea (mg/dl)	45.4 ± 20.2
Creatinine (mg/dl)	1.1 ± 0.3
Albumin (g/L)	43.3 ± 2.1
Alanine aminotransferase—ALT (U/L)	24.0 ± 14.4
Aspartate aminotransferase—AST (U/L)	19.8 ± 4.6
White blood cell—WBC (10^3^/μl)	8.0 ± 1.5
Hemoglobin—Hb (g/dl)	13.8 ± 1.8

* Years from diagnosis, Data are means ±SD

Since the daily PES treatment was performed by the patients a home, compliance with treatment regimen was monitored by patient diaries, and by telephone interviews few times a week. Eight of the 11 patients received all 14 treatments, and three patients received 12 treatments.

No hypoglycemic episodes, other adverse events, or side effects were attributed to the treatment. PES treatment was found to be well tolerated and safe for daily use. However, it is necessary to confirm these findings in a longer study.

### Fasting State Analysis

**Fig 2:**
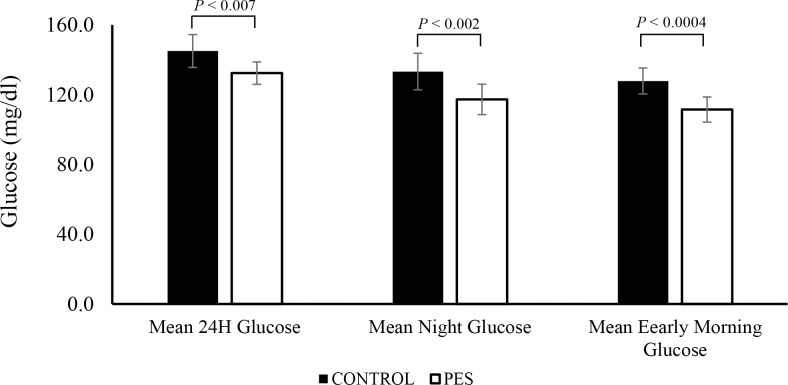
Mean interstitial glucose levels averaged over selected time intervals. 24 h, nighttime, and early morning glucose. CONTROL, Glucose average over the two weeks of control period, PES, Glucose average over the second week of PES treatment. Data are means ± SEM

Fig 2 and Table 2 show the mean interstitial glucose levels, averaged over selected time intervals of the day: mean 24 h, beginning at midnight, mean nighttime glucose from 01:00 hours to 05:00 hours, and mean early morning glucose averaged from 05:00 hours to 07:00 hours. Although specific meal times were encouraged, we found for each subject, variations in meal times, with different duration between meals. Therefore, those specific time intervals were chosen in order to reduce the effect of these variations. As can be seen, during the PES treatment period, the mean 24 h glucose was decreased significantly (*P* < 0.007). The most significant effect was on nocturnal glucose control (*P* < 0.0004). The mean daily glucose levels were also decreased although it did not reach clinical significant (*P* = 0.07). Average pre-meal glucose levels, calculated 1 h before meals, were also significantly reduced during the PES period as shown in Table 2. These results may suggest that most prominent decrease in plasma glucose was at the time intervals where hepatic glucose production (HGP) is most dominant.

**Table 2:** Fasting and Postprandial States Results (CGM data).

	CONTROL	PES	*P*-value
n	5	6	
Mean early morning glucose (mg/dl)	127.8 ± 7.4	111.5 ± 7.2	0.0004
Mean day glucose (mg/dl)	152.9 ± 9.8	139.7 ± 6.4	0.07
Mean night glucose (mg/dl)	133.2 ± 10.5	117.3 ± 8.7	0.002
Mean 24 h Glucose (mg/dl)	145.0 ± 9.4	132.3 ± 7.4	0.007
SD 24 h (mg/dl)	35.5 ± 2.5	30.7 ± 2.7	0.04
Before breakfast (mg/dl)	136.6 ± 6.8	119.7 ± 7.7	0.002
Before lunch (mg/dl)	129.7 ±8.5	112.3 ±6.8	0.02
Before dinner (mg/dl)	133.4 ± 8.6	117.7 ± 5.6	0.02
After breakfast (mg/dl)	166.6 ± 11.7	149.1 ± 9.2	0.02
After lunch (mg/dl)	169.9 ± 12.2	167.0 ± 13.1	0.1
After dinner (mg/dl)	167.0 ± 13.1	149.8 ± 10.5	0.1
Peak postprandial breakfast (mg/dl)	202.4 ± 11.9	185.5 ± 10.0	0.002
Peak postprandial lunch (mg/dl)	196.3 ± 12.1	176.5 ± 11.8	0.07
Peak postprandial dinner (mg/dl)	187.4 ± 11.7	177.3 ± 12.3	0.3

Notes: Before–mean BG 1 h before meal; After–mean BG over the 3rd hour after meal.

FBG = fasting blood glucose, MGTT = meal glucose tolerance test, AUC = area under the curve Data are means ±SEM

**Fig 3:**
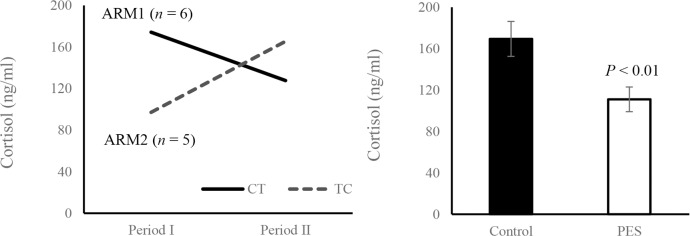
Cortisol levels at the end of the PES and control periods. a. The cortisol outcome of the two-period crossover trial presented for the CT (n = 6) and TC (n = 5) arms. CT: Control-Treatment arm, TC: Treatment-Control arm. b. Mean Cortisol levels (n = 11), Data are means ± SEM.

There was no significant difference in fasting serum insulin concentrations before and after the treatment session (CONTROL: 3.8 ± 0.6 and PES: 3.6 ± 0.5 μIU/ml, *P* = 0.6). However, fasting glucose (CONTROL: 151.5 ± 12.6 and PES: 125.3 ± 9.5 mg/dl, *P* < 0.002) and serum cortisol levels (CONTROL: 169.4 ± 16.9 and PES: 111.0 ± 11.9 ng/ml, *P* < 0.01) (Fig 3) were significantly decreased after two weeks of treatment. Serum glucagon concentrations were below the detection level of the test (< 2.5 pg/ml) in both CONTROL and PES. Mean HbA1c levels were significantly lower at the end of the study (7.3 ± 0.2 and 6.8 ± 0.2, *P* < 0.003). Plasma concentrations of triglyceride (142.6 ± 50.9 and 138.4 ± 50.9 mg/dl, *P* = 0.2), HDL-cholesterol (41.5 ± 10.7 and 38.4 ± 12.9 mg/dl, *P* = 0.4), and LDL-cholesterol (88.3 ± 28.9 and 95.4 ± 21.7 mg/dl, *P* = 0.3) were not significantly changed during the study.

### Postprandial Response Analysis

To evaluate the effect of PES on postprandial glycemia, an MGTT was performed at the end of both treatment and control sessions. There was no significant difference in the peak glucose and the area under the curve (AUC) during 2 h MGTT in the control period compared with the results at the end of the treatment period (82.4 ± 11.1 vs. 79.1 ± 8.3 mg/dl h, *P* = 0.4).

The postprandial state, defined as the 3rd hour period following ingestion of a meal, was evaluated, and the mean postprandial levels following breakfast, lunch and dinner were calculated. As can be seen in Table 2, there was a significant reduction (*P* < 0.02) only in postprandial breakfast levels, which was the "lighter" meal during the day.

However, some improvement was observed regarding glucose levels within the high range. A reduction in the mean AUC_180/iAUC percentage was observed during the treatment period, as obtained from the CGM data (30.2 ± 8.3 vs. 13.1 ± 5.0%, *P* < 0.01). Average peak postprandial glucose level was detected, within 4 h after meal ingestion, as shown in Table 2. During the PES period, mean peak postprandial breakfast glucose concentration was significantly decreased (P < 0.002). There was no significant change in mean peak postprandial lunch, and dinner glucose concentrations. These results may indicate that two weeks of PES treatment do not substantially affect peripheral glucose utilization.

### Multi-parametric Analysis

Currently, there are no standardization or reference targets for glycemic variability, and for combinations of CGM measures [36]. Therefore, due to the relatively short duration of treatment (2 weeks), we used a heat map analysis, which is an effective tool to capture trends, and correlations between glycemic parameters.

**Fig 4:**
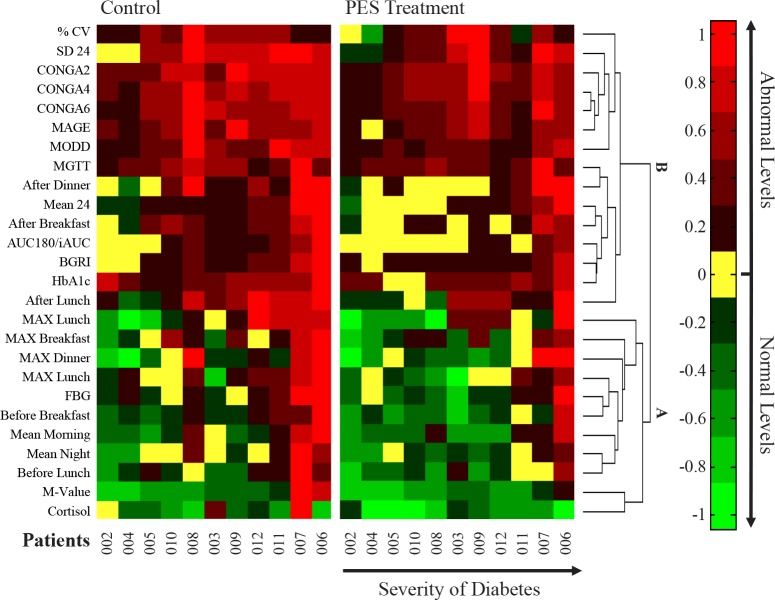
Multi-parametric, heat map analysis. Each column represents a single subject, and the color scale indicates the normalized value of each one of the parameters. The parameters are ordered by hierarchical clustering using the Euclidean distance metric algorithm. Cluster (A) parameters are related to mechanisms that involve HGP pathways, where cluster (B) parameters are more influenced from glucose utilization. More details are found in S2 and S3 Tables.

Fig 4 shows a multi-parametric heat map analysis, summarizing the calculated parameters extracted from the CGM data, as well as laboratory measures. The CGM CONTROL values were calculated over the two weeks of control period. Since there was a cumulative effect of the repeated treatments with respect to glucose levels, the CGM PES values were calculated over the second week of the treatment period, as glucose levels approached a new steady state, for comparison with the control period results.

Each column represents a single subject, and each row represents a normalized parameter. The heat map colors are in the green-red scale, where green is the estimated normal range of each one of the parameters, for example, the average morning glucose interval is [70 126]. Red represents abnormal levels, with brighter red for higher values. Patients and parameters are sorted according to severity of the disease, using the Euclidean distance metric clustering algorithm. Full details are found in S2 and S3 Tables.

It can be observed that the PES treatment tends to normalize (shifted to the yellow/ green bands), or improve (shifted to a darker red band) the parameter levels. The parameters, that are shown to have the largest improvement in most patients, are related to mechanisms that involve HGP pathways (cluster A), rather than glucose utilization (cluster B).

## Discussion

In this study, the effect of PES on blood glucose regulation in T2DM patients was evaluated in a prospective, randomized, crossover trial. The novel finding of this study is that repeated daily PES treatment may suppress endogenous glucose production, and thus can improve basal overnight and fasting glucose concentrations.

In T2DM patients, basal glucose concentrations are strongly associated with increased rates of glucose production [3]. The results of this study demonstrate an improvement in nocturnal glucose control, as well as a reduction in mean glucose levels before meals. On the other hand, there was no statistically significant difference in glucose tolerance evaluated by using the MGTT, serum insulin levels, and postprandial glucose excursion. These observations suggest that PES treatment may modulate HGP.

Several studies demonstrated an improvement in glucose tolerance after 30–90 min of EA treatment in rodent models of Type 1 and Type 2 diabetes. Due to their relatively long stimulation duration in relative small animals, these observations can be attributed, at least in part, to muscle contraction, which directly influence skeletal muscle and adipose tissue glucose consumption. Thus, in these cases, the effect of EA on insulin levels [37, 38], expression of GLUT4 [15, 39] and serum Free Fatty Acids (FFA) [40] can be regarded as similar to the effect achieved due to voluntary exercise [41–43]. In this study, muscle contraction mediated effect is negligible due to the short (5 min) duration of the stimulation and due to the low stimulation intensity. In addition, there was no significant effect on serum insulin levels and therefore our observations support the hypothesis that the primary target of PES is the autonomic nerves system (ANS) [44]. However, it is still possible that a longer duration of treatment, more than two weeks, may have additional beneficial effect on insulin sensitivity through restoration of normal sympathetic-parasympathetic balance [23, 28, 45].

In the current study, serum fasting cortisol levels, were decreased substantially after two weeks of treatment, and were correlated with fasting glucose levels. Cortisol is a glucocorticoid hormone, secreted by the adrenal cortex, and plays a key role in regulation of glucose homeostasis. Cortisol upregulates glucose production through gluconeogenesis, and inhibits glucose uptake and fatty acid mobilization in skeletal muscle and white adipose tissue [46, 47]. Although the mechanism of action is not fully understood, hypercortisolism was found to be associated with chronic metabolic complications such as insulin resistance, T2DM, dyslipidemia, and hypertension [48, 49]. Recently, cortisol regulation has been suggested as a new treatment strategy for T2DM through 11b-HSD1 enzyme inhibition [50]. The reduction in cortisol levels strengthen our assumption that a regulation of hepatic glucose gluconeogenesis appears to be involved in this treatment and the effect of PES in diabetes patients may be attributed, at least in part, to the Hypothalamic-Pituitary-Adrenal (HPA) axis.

The current study has several strengths but also a few limitations. We applied a cross over design and used continuous glucose monitoring in addition to discrete blood glucose measurement. CGM provides comprehensive information regarding glucose fluctuations during days and nights, as well as detection of safety events. For example in diabetes patients, the increase in glucose levels from nocturnal nadir until pre-breakfast level, due to the dawn phenomenon can be detected [51]. It should be clarified that this study was designed as a pilot study with obvious limitations that are related to the relative small sample size and the short duration of the intervention.

In conclusion, PES treatment may suppress HGP, and thus, act to maintain basal overnight and fasting glucose concentrations. This effect may be mediated, at least in part, via hypothalamic-pituitary-adrenal axis modulation. The treatment is noninvasive and can be easily performed by the patients at home with minimal discomfort. Future studies, including larger populations, and longer follow up periods, are needed to gain better understating of whether these methods can become a part of a standard clinical care. Once proven over a larger cohort of patients, PES may provide an additional potential treatment strategy for the current armada available for T2DM patients.

## Supporting Information

S1 CONSORT Checklist Consort checklist (PDF)

S1 Protocol Study protocol (PDF)

S1 Table Continuous Glucose Analysis–Definitions and measures of glucose series (PDF)

S2 Table Multi-Parametric Heat Map Analysis—Parameter values normalization table. (PDF)

S3 Table Experimental Data (PDF)
