# Supplementary material for: Effect of Peripheral Electrical Stimulation (PES) on Nocturnal Blood Glucose in Type 2 Diabetes: A Randomized Crossover Pilot Study
Source: PLoS One. 2016 Dec 20;11(12):e0168805. doi: 10.1371/journal.pone.0168805 (PMC5173375; doi:10.1371/journal.pone.0168805)
Supplement: S1 Protocol — (PDF) [file pone.0168805.s002.pdf]

S1: Clinical Study Protocol

Clinical Trial Protocol No. CTP: PES-TAU-001

EFFECT OF SHORT PERIPHERAL ELECTRICAL STIMULATION (PES) ON BLOOD GLUCOSE LOWERING ACTION IN TYPE-II DIABETES PATIENTS

Investigators	*Name*	*Contact information*
**Principle Investigator**	DR. Shai Efrati	Phone: +972-(0)8-977-9393/5<br>Cell : +972-549-212-866<br>Email : efratishai@013.net
Investigator	Prof. Eshel Ben-Jacob	Email : eshelbj@gmail.com
Investigator	Merav Catalogna	Email : merav.catalogna@gmail.com
Investigator	DR. Uri Nevo	Email : nevouri@post.tau.ac.il
Investigator	DR. Keren Doenyas	Email :kerendoenyas@gmail.com

Table of Contents

TABLE OF CONTENTS II
LIST OF ABBREVIATIONS III
STUDY SUMMARY 1
1 INTRODUCTION 2

1.1 BACKGROUND 2
1.2 TREATMENT RATIONALE AND RISK 2

2 STUDY OBJECTIVES 3

2.1 PRIMARY OBJECTIVE 3
2.2 SECONDARY OBJECTIVES 3

3 STUDY DESIGN 3

3.1 GENERAL DESIGN 3
3.2 PRIMARY OUTCOME MEASURES 5
3.3 SECONDARY OUTCOME MEASURES 5

4 SUBJECT SELECTION AND WITHDRAWAL 6

4.1 INCLUSION CRITERIA 6
4.2 EXCLUSION CRITERIA 6
4.3 SUBJECT RECRUITMENT AND SCREENING 6
4.4 EARLY WITHDRAWAL OF SUBJECTS 6

5 STUDY PROCEDURES 7

5.1 SCREENING VISIT 7
5.2 RANDOMIZATION 7
5.3 TRIAL VISIT 1 (DAY 1)/ 4 (CROSSOVER) (DAY 29) 7
5.4 TRIAL VISIT 2 (DAY 8) / 5 (DAY 29) 8
5.5 TRIAL VISIT 3 (DAY 12) / 6 (DAY 40) 8
5.6 FOLLOW-UP VISIT (DAY 57) 8
5.7 HOME-CARE TREATMENT AND SUBJECT COMPLIANCE MONITORING 9

6 STUDY INTERVENTIONS 10

6.1 PES TREATMENT 10

6.1.1 Active Treatment protocol 10
6.1.2 Receipt and return of homecare device 11

7 STATISTICAL ANALYSIS 11

7.1 SAMPLE SIZE DETERMINATION AND SUBJECTS 11
7.2 STATISTICAL METHODS 11

8 ADMINISTRATION AND REGULATION 12

8.1 INFORMED CONSENT 12
8.2 CONFIDENTIALITY 12
8.3 STUDY FILES 12
8.4 ADVERSE EVENTS 12
8.5 ASSESSMENT OF ADVERSE EVENTS 13
8.6 SERIOUS ADVERSE EVENTS 13

9 ETHICAL CONSIDERATIONS 14
10 REFERENCES 14

List of Abbreviations

AE	Adverse Event
BMI	Body Mass Index
CGM	Continuous Glucose Monitoring
CRC	Clinical Research Center
CRF	Case Report Form
DC	Duty Cycle
FBG	Fasting Blood Glucose
FFP	Functional Focal Point
HbA1c	Glycated Hemoglobin
HDL	High-Density Lipoprotein
HOMA	Homeostatic Model Assessment
IFG	Impaired Fasting Glucose
IGT	Impaired Glucose Tolerance
LDL	Low-Density Lipoprotein
PES	Peripheral Electrical Stimulation
SAE	Serious Adverse Event
TENS	Transcutaneous Electrical Nerve Stimulation
TG	Triglyceride

Study Summary

Title	Effect of short peripheral electrical stimulation (PES) on blood glucose lowering action in type-II diabetes patients
Protocol Number	CTP: PES-TAU-001
Methodology	Pilot study, Interventional, Open label, Randomized, Crossover trial
Endpoint Classification	Safety/Efficacy Study
Study Duration	6 weeks
Study Center	Single-center
Objectives	Evaluation of safety, tolerability, and glucose lowering effect of short duration peripheral electrical stimulation (PES) applied non-invasively at skin functional focal points (FFP) in type-II diabetes patients.
Estimated Enrollment	10 patients
Diagnosis and Main Inclusion Criteria	Type II diabetes, stable treatment regimen for at one month prior to randomization, Age 18-75, BMI < 35 Kg/m^2
Structure	2 Arms: (1) Treatment period/ Control period (2) Control period/ Treatment period
Study Product, Dose, Route, Regimen	Eligible volunteers will receive daily PES treatment for 5 minutes for two weeks. Device: Intellistim, BE-28TC, Frequency 1.33Hz/ bursts 16Hz rectangular bi-phasic, Pulse width: 150µs
Safety	The device and the electrodes used are CE approved.
Duration of administration	2 weeks
Reference therapy	No treatment

1 Introduction

This document is a protocol for a human research study. This study is to be conducted according to Israel and international standards of Good Clinical Practice, applicable government regulations and Institutional research policies and procedures.

1.1 Background

Diabetes mellitus is a one of the most widespread health problems in modern times, and was recognized by the United Nations as a global epidemic [1]. With the growing prevalence and increasing attention to the challenges of treatment, prevention of diabetes is a key issue in public health. Although it is well established that the risk of developing diabetes mellitus, may be reduced through lifestyle modifications [2], in practice, most patients show poor adherence to therapeutic exercise-training regimens because of a busy lifestyle or a medical disability.

We have recently demonstrated (in preparation for publication) that noninvasive peripheral electrical stimulation (PES) treatment of a very short duration (2-3 min) may stimulate glucose utilization and improve hepatic insulin sensitivity in rats. This improvement was reflected by increasing the efficacy of cellular glucose uptake and enhancement of glycogen synthesis, as evaluated using hyperinsulinemic-euglycemic clamp test. We have also showed that repeated PES treatment (3 weeks, 3 times/wk), significantly inhibited the progression of glucose intolerance in normal and insulin-resistant rats and prevented HFD-induced gains in body weight and fat mass. This treatment may offer a new intervention approach for diabetes, as a part of homecare program, with no or minimal discomfort.

1.2 Treatment Rationale and Risk

The rational for the selection of treatment time, a single treatment duration, and a treatment session duration is based on prior knowledge from our related animal models (in preparation for publication). However, several clinical trials were conducted using external electrical stimulation devices (TENS, electro-acupuncture) have shown safety with more intense treatment protocol. For example, pain management in labor [3], treatment of women with Polycystic Ovary Syndrome [4] and treatment of diabetic neuropathy [5]. The stimulation device is CE approved for the use with much longer time periods, time sessions and higher intensities. A description of the stimulation device characteristics and safety data is detailed section 6.1.3.

2 Study Objectives

The aim of this study is to evaluate safety, tolerability, and the glucose-lowering effect of noninvasive peripheral electrical stimulation (PES) as an alternative treatment for diabetes.

2.1 Primary Objective

(1) To assess the safety and tolerability of an acute, and two weeks treatment of PES in diabetes patients.

(2) To assess the effect of PES treatment applied non-invasively on blood glucose levels in diabetes patients.

2.2 Secondary Objectives

(1) To investigate whether two weeks of PES treatment may reduce glucose levels, and glucose variability.

3 Study Design

3.1 General Design

a. Study type: Interventional
b. Study design: Open label, Randomized, Parallel Assignment
c. Endpoint Classification: Efficacy Study/ safety
d. Single center
e. Duration of administration: 2 weeks
f. General procedure: Eligible volunteers will receive daily PES treatment for 5 minutes at the morning, for two weeks. Device: Intellistim, BE-28TC, Frequency 1.33Hz/ bursts 16Hz, DC 33% rectangular bi-phasic, Pulse width: 150µs. Daily use of continuous glucose monitoring throughout the study.

A general schematic diagram of the study design is illustrated in Fig. 1.

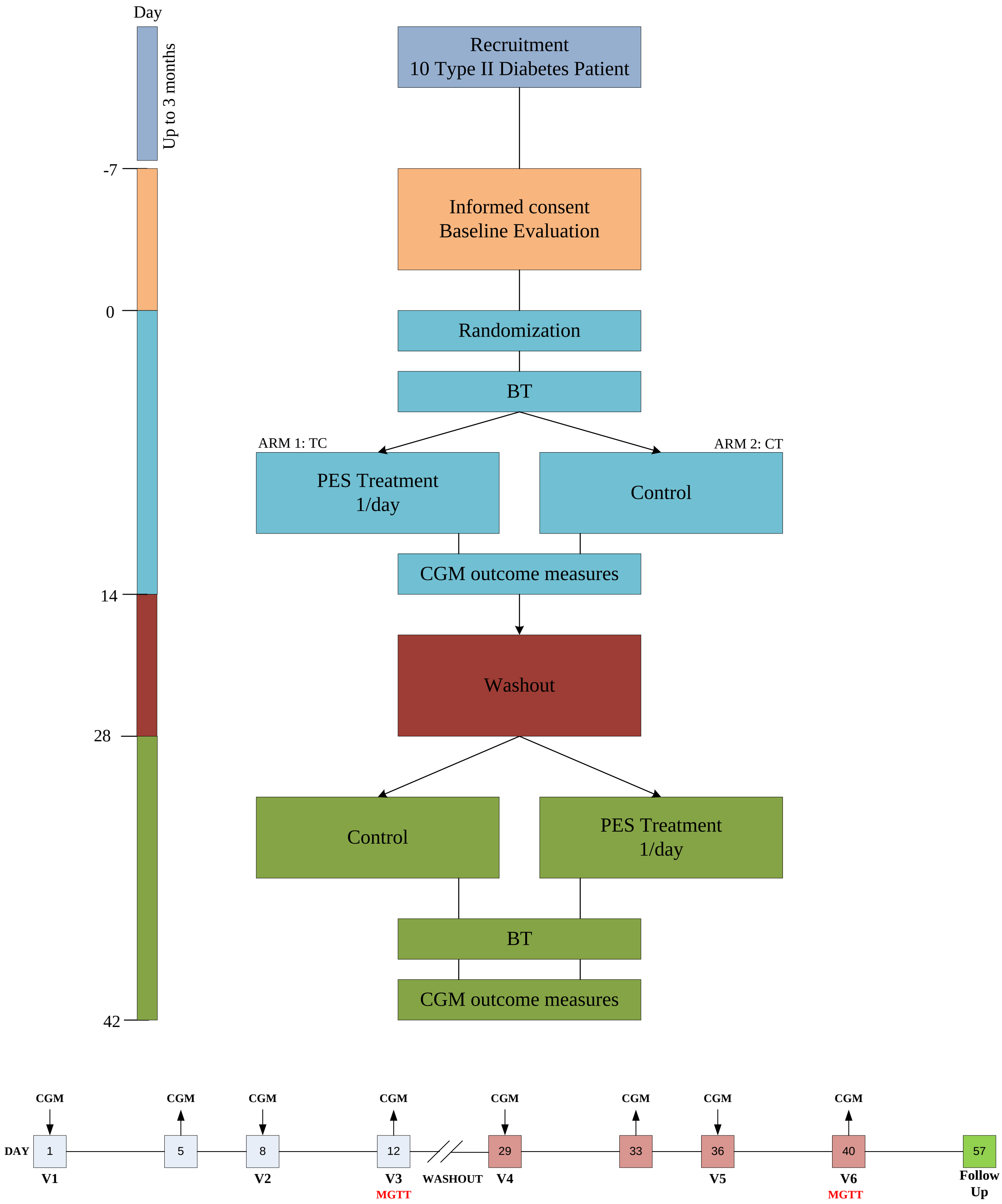

*Fig. 1. **A**. Trial design – General schematic diagram. PES – peripheral electrical stimulation, CGM – continuous glucose monitoring, BT – blood tests. **B** Visits and procedures. MGTT – meal glucose tolerance test*

3.2 Primary Outcome Measures

a. Number of participants with hypoglycemia, and/ or adverse events that are related to treatment [Time Frame: Baseline through 2 months] [Designated as safety issue: Yes]

3.3 Secondary Outcome Measures

a. Incidence of minor side effects related to treatment* [Time frame: Baseline through 2 months] [Designated as safety issue: No]
b. Changes in mean interstitial glucose levels** measured by CGM [[Time frame: Baseline, 1, 2, 5, 6 weeks] [Designated as safety issue: No]
c. Changes in meal glucose tolerance (MGTT) [Time Frame: 0, 2, 5 weeks] [Designated as safety issue: No]

* Possible related side effects:

1. Pain/ uncomfortable sensation during PES treatment.
2. Skin assessment (areas of skin under the electrodes will be assessed for redness, tenderness, or any other change in skin condition)

** Changes in mean interstitial glucose levels includes:

3. Blood glucose levels: before and two hours after meals.
4. Mean Glucose levels (mean 24 h, daytime, and nighttime
5. Percentage of Glucose Levels >180 mg/dl at day time/night time
6. Glucose Variability Measure at daytime and nighttime

4 Subject Selection and Withdrawal

The study will include 10 type-II diabetes patients. Patients should be on a stable diabetes treatment regimen for at least three months prior to randomization, and will be required to stay on the same regimen during the study.

4.1 Inclusion Criteria

a. Patients diagnosed with type II diabetes with fasting blood glucose >126 mg/dl, for at least one year prior to randomization
b. Age 18-75 years old.
c. Body mass index BMI < 35 Kg/m^2
d. Stable glucose lowering drugs regimen for at least one month prior to randomization
e. Subjects are capable of giving informed consent

4.2 Exclusion Criteria

a. Pregnancy, or nursing
b. 10< HbA1c < 6
c. Patients with acute myocardial infarction or stroke during the last 6 month before their inclusion.
d. Permanent pacemakers
e. Metal prosthesis
f. Resting blood pressure > 160/ 100 mmHg
g. Skin disease
h. Known allergy to metals.
i. Creatinine >1.5 mg/dL or eGFR<60
j. Any significant abnormalities in liver enzymes, electrolytes, thyroid function and hematologic evaluation.
k. Treatment with steroids or beta-blockers treatment with psychiatric medications
l. Known HIV, HCV or HBV infection.
m. Change in blood lowering medications during the study/ follow-up
n. Low compliance to the study protocol.
o. Withdrawn consent

4.3 Subject Recruitment and Screening

Patients with T2DM will be recruited for this study during routine clinical visits at the Assaf Harofeh Medical Center. Prior to subject participation in any screening procedures, the investigator will explain the nature and purpose of the study to eligible patients and written informed consent will be obtained prior to the performance of any study-related procedures. Patients willing to sign the informed consent will be recruited to the study. Participants will be recruited over 3 months and therefore, the complete duration of the study is expected to be 4-5 months, during which time at least 10 patients will be enrolled. Screening procedures according to selection criteria will be performed during screening visit as details in sections 5.1.

4.4 Early Withdrawal of Subjects

A subject may be withdrawn from the study prior the expected completion due to the following reasons:

(1) Safety reasons

(2) Failure of subject to adhere to protocol requirements: homecare treatments, disease progression, etc.

5 Study Procedures

5.1 Screening Visit

The purpose of the screening process is to identify and verify eligible participants through achieving the objectives of an informed consent process, complete baseline measurements and procedures, and randomize participants into the study arms. All eligibility and baseline data must be reviewed prior to scheduling randomization.

(1) A preliminary screen will be done to determine eligibility either by phone or at the clinical site prior to the screening visit. Volunteers diagnosed with type II diabetes in the age group of 18-75 years will be invited for a screening visit.
(2) During the screening visit, potential participants will be provided with information about the study, their questions will be answered, and they will receive a consent form to review with clinic staff.
(3) After signing informed consent:

   a. Interview: demographic data, details of family history of diabetes, hypertension and cardiovascular diseases, physical activity, diet habits and details of medications.
   b. Physical examination, temperature, body weight, height and Abdominal circumfluence
   c. ECG and blood pressure
   d. Blood sample will be taken for complete blood count and chemistry including electrolytes, liver and renal functions, TSH and HbA1c.

(4) Patients will be instructed to continue their current treatment throughout the study.

5.2 Randomization

Eligible participants will be randomized to the two arms of the study within 1:1 rate: Treatment-Control (TC) arm (n=5), and Control-Treatment (CT) arm (n=5). Both groups will be subjected to the same procedures but in cross-mach order. Randomization will be performed according to a computer-generated randomization numbers.

5.3 Trial Visit 1 (day 1)/ 4 (Crossover) (day 29)

During the first trial visit, patients will be subjected to the following procedures:

(1) Registration at 7:00 AM.
(2) All patients will undergo comprehensive clinical and laboratory baseline data evaluation: physical examination, body weight, height and abdominal circumfluence, ECG, temperature and blood pressure measurements.
   Laboratory tests will be performed after 8-hours overnight fasting (water is allowed). Blood will be taken for the following tests: blood counts, fasting blood glucose, HbA1c, lipid profile, liver and renal function.
(3) PES treatment (TC arm) as described in section 6. Patients will receive detailed instructions for homecare therapy.
(4) Insertion of continuous glucose monitor (CGM) sensor (Abbott, Freestyle Navigator). Patients will be educated on proper use and calibration of the CGM. Patient will be asked to stay at the CRC for 2 hours after sensor insertion for initial calibration.
(5) Patients will be instructed on how to record their daily meals and physical activities.
(6) Patients will be instructed to remove the CGM after 122 hours of use (day 6, morning).

5.4 Trial Visit 2 (day 8) / 5 (day 29)

Both TC and CT Arms will be invited to trial visit 2, seven days after initiation of the trial.
Patients will be subjected to the following procedures:

(1) Registration and interview.
(2) Clinical and laboratory evaluations: physical examination, body weight and abdominal circumfluence, ECG, temperature and blood pressure measurements.
Laboratory tests will be performed after 8-hours overnight fasting (water is allowed). Blood will be taken for the following tests: blood count, fasting blood glucose, lipid profile, liver and renal function.
(3) Areas of skin under the electrodes will be assessed and photo (TC arm).
(4) Replacement of CGM sensor and data downloading.

5.5 Trial Visit 3 (day 12) / 6 (day 40)

Both TC and CT Arms will be invited to trial visit 3, 12 days after initiation of the trial.
Patients will be subjected to the following procedures:

(1) Registration and interview.
(2) Clinical and laboratory evaluations: physical examination, body weight and abdominal circumfluence, ECG, temperature and blood pressure measurements.
Laboratory tests will be performed after 8-hours overnight fasting (water is allowed). Blood will be taken for the following tests: blood count, fasting blood glucose, lipid profile, liver and renal function, (HbA1c at visit 6).
(3) Areas of skin under the electrodes will be assessed and photo (TC arm).
(4) One hour after the treatment, breakfast (Ensure Plus, Abbott -355 calories/50 g carbohydrate) will be provided.
Blood glucose levels will be monitored prior to breakfast (both arms) and then continue every 30 min for two hours. 2ml blood will also be collected for insulin, C-peptide, Glucagon and Cortisol evaluation every 60 min for two hours.
Glucose will be provided if blood glucose level will be < 65 mg/dL (amount and way of administration: oral or IV - will be determined by the investigator). Regular insulin 0.1IU/kg will be provided if blood glucose level will be >350 mg/dL.
(5) Patient will be discharged from CRC at 15:00. Safety assessments will be performed prior to discharge: ECG, BP. Areas of skin under the electrodes will be assessed and photo (TC arm).
(6) CGM sensor data downloading.

5.6 Follow-up Visit (day 57)

All patients will be invited for a follow -up visit, 2 weeks after the end of the clinical trial. During that visit the following procedures will be done:

(1) Registration and interview.
(2) Clinical and laboratory evaluations: physical examination, body weight and abdominal circumfluence, ECG, temperature and blood pressure measurements.
Laboratory tests will be performed after 8-hours overnight fasting (water is allowed). Blood will be taken for the following tests: blood count, fasting blood glucose, HbA1c, insulin and C-peptide, lipid profile, liver and renal function.
(3) Areas of skin that was under the electrodes will be assessed and photo (both arms).

5.7 Home-care treatment and subject compliance monitoring

Home-care treatment - During 2 weeks treatment session, patients will be subjected to a home-care treatment regimen. Patients under treatment will be instructed to use the device daily between 7:00 and 8:30 AM, 30-60 minutes before breakfast for 5 minutes. They will also be instructed to use the intensity and therapeutic program as determined during the previous trial visit. For safety reasons, this information will be locked by the study member during the treatment visit.
Home visits may be considered for participants who have a problem using the device and/ or to provide an opportunity to encourage them to perform the treatment regularly.
Patients will be asked to fill out a food diary throughout the glucose monitoring periods, and will also be asked to record information about intense physical activity, stressful event, illness, missed medications etc.

Alarms
FreeStyle Navigator system will be set to alert users at the following conditions:

1. A Low glucose threshold alarm: BG < 60 mg/dl
2. A High glucose threshold alarm: BG > 350 mg/dl
3. Time of Calibration alarm
4. A Low battery alarm
5. A data loss alarm warns: Occur when the sensor has expired, when the transmitter-receiver connection is broken, when a calibration has expired or when the sensor is not working properly.

Hypoglycemic events (BG < 60 without symptoms or BG < 80 with hypoglycemic symptoms) at home setting should be reported directly to the investigator. Modification of the anti-diabetes medication regimen will be considered after two hypoglycemic events.

Subject compliance monitoring - The study team will assess and track subject compliance with the study treatment regimen. 5-7 times a week (during the homecare therapy day), individual contact person will collect data by telephone interview, on reaction to treatment, appetite, diet and exercise according to the follow-up questionnaire. Feedback will be given to participants according to the subject's compliance and potential side effects. Treatment compliance will be also electronically monitored by downloading information of the treatment time recorded within the device during the trial visits.

6 Study Interventions

6.1 PES Treatment

6.1.1 Active Treatment protocol

Treatment functional focal points (FFP) will be located by trained study member during the first treatment visit. FFPs selected for the active treatment arm will be located bilaterally in the lower extremities (corresponding to ST-36 acu-point).
Location: On the anterior aspect of the lower extremities, for fingers width below the kneecap, and one finger-breadth lateral to the anterior crest of the tibia (Fig.4). Selected FFPs will be marked using a permanent pen.

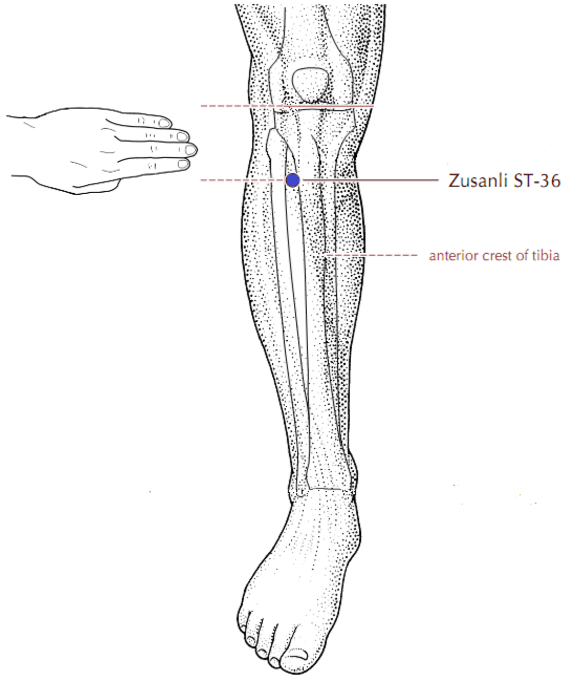

Fig. 4. Location of FFP related to Active treatment arm.*

Self adhesive TENS electrodes ∅25mm will be placed on both FFSs after cleaning the area with alcohol. The treated area should be dry, unbroken, and not infected, or inflamed. The positive (red) wire will be connected to the right electrode and the negative (black) wire will be connected to the left electrode. The intensity of the electrical stimulation should be adjusted individually to produce mild local muscle contractions, without pain or discomfort. Current will be applied for 5 minutes.

Electrical characteristics:
Device: BEAC Biomedical Intellistim BE-28TC

Pulse shape: bi-phasic, square wave
Signal pattern: 1.33 Hz with burst pulses frequency of 16 Hz and DC of 33%.
Output current: max 130mA peak value, <10mA average over 500 ohm load, 20 intensity levels (intensity is adjusted individually).

* The figure is based on "Deadman, Peter, Mazin Al-Khafaji, and Kevin Baker. A manual of acupuncture. East Sussex, UK: Journal of Chinese Medicine Publications, 1998".

6.1.2 Receipt and return of homecare device

Devices will be obtained for homecare use after the first treatment visit. Participants will receive comprehensive information about device usage, as well as instruction manual in Hebrew. Participants will also receive 10 adhesive electrodes (∅ 25 mm) and a pair of AA 1.5V batteries. Any damaged or unusable device will be documented in the study files.
At the completion of the study, homecare devices and unused electrodes will be returned to the study team members.

7 Statistical Analysis

7.1 Sample Size Determination and Subjects

Since this study was primarily designed as a pilot study for the assessment of tolerability and safety of the treatment procedure, no formal sample size calculation was performed. Based on experience with similar studies in the past, a group of 10 patients was considered to provide sufficient observation data, taking into account early withdrawal of subjects or non-valuable subjects in a rate of 10-30%.

7.2 Statistical Methods

Endpoints data were evaluated by using the crossover analysis of variance as described by Jones and Kenward {Jones, 1989 #1066}. Comparisons between treatment and control periods will performed using the paired nonparametric Wilcoxon signed rank test or the paired student's t-test with two-tail distribution when a normality assumption holds according to Kolmogorov-Smirnov test. General metabolic parameters mean values between the groups will be compared by Student's t-test analysis (unpaired, two-tailed). A value of $p \leq 0.05$ is considered significant. Statistical analysis will be performed using Matlab v.7.1 Statistics Toolbox.

8 Administration and regulation

8.1 Informed Consent

The investigator will obtain written informed consent from the patient participates in this study after adequate explanation of the aims, methods, objectives, and potential hazards of the study and prior to undertaking any study-related procedures. The investigator must utilize a consent form for documenting written informed consent. Informed consent will be appropriately signed and dated by the patient or the subject's legally authorized representative and the person obtaining consent.

8.2 Confidentiality

Patients' anonymity will be strictly maintained and that their identities are protected from unauthorized parties. The information is not to be disclosed to any third party (except for medical stuff or employees or agents directly involved in the conduct of the study or as required by law).

8.3 Study Files

The medical records will be maintained adequately to enable good data storage and latter on management. Subject clinical source documents would include (although not, limited to) the following: subject hospital/clinic/ hyperbaric unit records, physician's and nurse's notes, appointment book, original laboratory reports, electroencephalogram (EEG), X-ray, SPECTs, CT and special assessment reports, consultant letters, screening and enrollment log etc.

8.4 Adverse Events

An adverse event (AE) is any untoward medical occurrence in a clinical investigation subject administered a medicinal product and which does not necessarily have a causal relationship with this treatment. An AE can therefore be any unfavorable and unintended sign, symptom, or disease temporally associated with the use of a medicinal product, whether or not considered related to the medicinal product. Pre-existing events, which increase in severity or change in nature during or as a consequence of use of a medicinal product in human clinical trials, will also be considered AEs.
Any medical condition or clinically significant laboratory abnormality with an onset date before the screening visit and not related to study procedures is considered to be pre-existing, and should be documented in the case report form.
Any AE (i.e., a new event or an exacerbation of a pre-existing condition) with an onset date after the screening visit up to the last day on study (including the follow-up, off study medication period of the study), should be recorded as an AE on the appropriate CRF page(s).
An AE does not include:

- Medical or surgical procedures (e.g. surgery, Endoscopy, tooth extraction, transfusion); the condition that leads to the procedure are an adverse event.

- Pre-existing diseases or conditions or laboratory abnormalities present or detected prior to the screening visit that does not worsen.

- Situations where an untoward medical occurrence has not occurred (e.g. hospitalization for elective surgery, social and/or convenience admissions).

- Overdose of either study drug or concomitant medication without any signs or symptoms unless the subject is hospitalized for observation.

8.5 Assessment of Adverse Events

All AEs will be assessed by the investigator and recorded on the appropriate CRF page, including the date of onset and resolution, severity, relationship to study drug or study procedures, outcome and action taken with study medication.
The relationship to study drug therapy or study procedures should be assessed using the following definitions:

No: Evidence exists that the adverse event has an etiology other than the study drug or study procedures (eg. pre-existing condition, underlying disease, intercurrent illness, or concomitant medication).

Yes: A temporal relationship exists between the event onset and administration of the study drug or between the event and the study procedures. It cannot be readily explained by the subject's clinical state or concomitant therapies and, in the case of the study drug, appears with some degree of certainty to be related based on the known therapeutic and pharmacologic actions or adverse event profile of the study drug. In case of cessation or reduction of the dose, the event abates or resolves and reappears upon re-challenge. It should be emphasized that ineffective treatment should not be considered as causally related in the context of adverse event reporting.
These criteria in addition to good clinical judgment should be used as a guide for determining the causal assessment. If it is felt that the event is not related to study drug therapy, then an alternative explanation should be provided.

8.6 Serious Adverse Events

A serious adverse event (SAE) is defined as follows:

Any adverse drug experience occurring at any dose that results in any of the following outcomes:

- Death;
- Life-threatening situation (subject is at **immediate** risk of death);
- In-patient hospitalization or prolongation of existing hospitalization (excluding those for study therapy or placement of an indwelling catheter, unless associated with other serious events);
- Persistent or significant disability/incapacity;
- Congenital anomaly/birth defect in the offspring of a subject who received study drug;

Other: medically significant events that may not result in death, be immediately life-threatening, or require hospitalization, may be considered a SAE when, based upon appropriate medical judgment, they may jeopardize the Subject and may require medical or surgical intervention to prevent one of the outcomes listed in this definition.
Examples of such events are:

- Intensive treatment in an emergency room
- Blood dyscrasias or convulsions that do not result in hospitalization
- Development of drug dependency or drug abuse

The investigator should notify the Institutional Review Board (IRB) or Independent Ethics Committee (IEC) as soon as is practical, of serious events in writing where this is required by local regulatory authorities, and in accordance with the local institutional policy.

9 Ethical Considerations

The protocol will be submitted to a properly constituted independent Ethics Committee (EC) or Institutional Review Board (IRB), for formal approval of the study conduct. The decision of the EC/IRB concerning the conduct of the study will be made in writing to the investigators prior to the initiation of the study.

All subjects for this study will be provided a consent form describing this study and providing sufficient information for subjects to make an informed decision about their participation in this study. See Attachment 2 for a copy of the Subject Informed Consent Form. This consent form will be submitted with the protocol for review and approval by the EC/IRB for the study. The formal consent of a subject, using the EC/IRB approved consent form, must be obtained before that subject undergoes any study procedure. The consent form must be signed by the subject or legally acceptable surrogate, and the investigator-designated research professional obtaining the consent.

10 References

1. *World Health Organization - Diabetes*. [Web Page] 2011 [cited 2011 March 13]; Available from: http://www.who.int/topics/diabetes_mellitus/en/.
2. Tuomilehto, J., et al., *Prevention of type 2 diabetes mellitus by changes in lifestyle among subjects with impaired glucose tolerance.* New England Journal of Medicine, 2001. **344**(18): p. 1343-1350.
3. Dowswell, T., et al., *Transcutaneous electrical nerve stimulation (TENS) for pain relief in labour.* Cochrane Database Syst Rev, 2009(2): p. CD007214.
4. Stener-Victorin, E., et al., *Low-frequency electroacupuncture and physical exercise decrease high muscle sympathetic nerve activity in polycystic ovary syndrome.* Am J Physiol Regul Integr Comp Physiol, 2009. **297**(2): p. R387-95.
5. Forst, T., et al., *Impact of low frequency transcutaneous electrical nerve stimulation on symptomatic diabetic neuropathy using the new Salutaris (R) device.* Diabetes Nutrition & Metabolism, 2004. **17**(3): p. 163-168.
