# Supplementary material for: Effect of Peripheral Electrical Stimulation (PES) on Nocturnal Blood Glucose in Type 2 Diabetes: A Randomized Crossover Pilot Study
Source: PLoS One. 2016 Dec 20;11(12):e0168805. doi: 10.1371/journal.pone.0168805 (PMC5173375; doi:10.1371/journal.pone.0168805)
Supplement: S1 Table — (PDF) [file pone.0168805.s003.pdf]

Supplementary Data

Supplementary S1 Table. Continuous Glucose Analysis – Definitions and measures of glucose series

Parameter	***Definition***	***Equation***	***Reference***
**Statistical measures and temporal events**			
**$MEAN_I$**	Mean glucose over time interval I<br>I: 24 h<br>Before Breakfast/ Lunch/ Dinner<br>After Breakfast/ Lunch/ Dinner<br>Day<br>Night<br>Early Morning	$MEAN_I = \frac{\sum_{i=1}^{N_I} g_i}{N_I}$	[1]
**AUC180/ iAUC**	*AUC180* **-** Percentage Area under glucose curve above target range (180 mg/dl)<br>*iAUC* - includes all area below the glucose curve and above the fasting concentration.	$\frac{\sum_{i=1}^{N_I-1}(g_i + g_{i+1} - 360) \cdot \Delta t/2,\ \ g_i \geq 180}{\sum_{i=1}^{N_I-1}\left(g_i + g_{i+1} - 2 \cdot g_{fasting}\right) \cdot \Delta t/2,\ \ g_i \geq g_{fasting}}$	[2]
**Max_I**	Max glucose level within 4 h after meal ingestion<br>I: Breakfast/ Lunch/ Dinner	$\max_{4\,h}[g_0\ ..\ g_{N_I}]$	

**Glucose variability and risk assessment**			
**SD**	Standard Deviation	$$SD = \sqrt{\frac{\sum_{i=1}^{N_I}(g_i - Mean_I)^2}{N_I - 1}}$$	[1]
**% CV**	Coefficient of Variation	$$CV = \frac{SD_I \cdot 100}{Mean_I}$$	[1]
**M-value**	weighted SD	$$M = \frac{\sum_{i=1}^{N_I}\left\|10 \cdot \log\frac{g_i}{120}\right\|^3}{N_I}$$	[3]
**MAGE**	Mean Amplitude of Glycemic Excursion	$$MAGE = MEAN\|\lambda\|$$ where<br>λ = change in glucose levels over each excursion from peak to nadir (or nadir to peak)<br><br>and $$\lambda > SD_{24}$$	[4]
**MODD**	Mean of Daily Differences	$$MODD = \frac{\sum_{i=1}^{N_I}\left\|(g_i - g_{i-f\cdot 24})\right\|}{N_I}$$	[5]
**CONGAn**	SD of the difference between values obtained exactly n hours apart<br>n = 1, 2, 4, etc.	$$CONGA_n = \sqrt{\frac{\sum_{i=1}^{N_I}(D_t - \bar{D})^2}{N_I - 1}}$$	[6]

		where<br>$$D_t = g_i - g_{i-f\cdot n}$$<br>and<br>$$\overline{D} = \frac{\sum_{i=1}^{N_I} D_t}{N_I}$$	
**BGRI**	LBGI - Low Blood Glucose Index<br>HBGI - High Blood Glucose Index<br>BGRI - Blood glucose risk index	$$f(g_i) = 1.509 \cdot [(\ln(g_i)^{1.084} - 5.381]$$<br>$$r(g_i) = 10 \cdot f(g_i)^2$$<br>$$rl(g_i) = r(g_i) \;\; if \;\; f(g_i) < 0 \; and \;\; 0 \quad o.w$$<br>$$rh(g_i) = r(g_i) \;\; if \;\; f(g_i) > 0 \; and \;\; 0 \quad o.w$$<br>$$LBGI = \frac{1}{N_I}\sum_{i=1}^{N_I} rl(g_i)$$<br>$$HBGI = \frac{1}{N_I}\sum_{i=1}^{N_I} rh(g_i)$$<br>$$BGRI = LBGI + HBGI$$	[7]

g_i = Continuous glucose monitoring (CGM) glucose time series – glucose reading i

N_I = number of glucose readings for a given time interval I

Δt = time interval between adjacent samples (min)

f = sampling frequency (samples per hour)

References

[1] Rodbard D (2007) Optimizing display, analysis, interpretation and utility of self-monitoring of blood glucose (SMBG) data for management of patients with diabetes. J Diabetes Sci Technol 1: 62-71

[2] Wolever TM (2004) Effect of blood sampling schedule and method of calculating the area under the curve on validity and precision of glycaemic index values. Br J Nutr 91: 295-301

[3] Schlichtkrull J, Munck O, Jersild M (1965) The M-value, an index of blood-sugar control in diabetics. Acta Medica Scandinavica 177: 95-102

[4] Baghurst PA (2011) Calculating the mean amplitude of glycemic excursion from continuous glucose monitoring data: an automated algorithm. Diabetes Technol Ther 13: 296-302

[5] Service FJ, Nelson RL (1980) Characteristics of glycemic stability. Diabetes Care 3: 58-62

[6] McDonnell CM, Donath SM, Vidmar SI, Werther GA, Cameron FJ (2005) A novel approach to continuous glucose analysis utilizing glycemic variation. Diabetes Technol Ther 7: 253-263

[7] Kovatchev BP, Clarke WL, Breton M, Brayman K, McCall A (2005) Quantifying temporal glucose variability in diabetes via continuous glucose monitoring: mathematical methods and clinical application. Diabetes Technol Ther 7: 849-862
