# Supplementary material for: Effect of Peripheral Electrical Stimulation (PES) on Nocturnal Blood Glucose in Type 2 Diabetes: A Randomized Crossover Pilot Study
Source: PLoS One. 2016 Dec 20;11(12):e0168805. doi: 10.1371/journal.pone.0168805 (PMC5173375; doi:10.1371/journal.pone.0168805)
Supplement: S2 Table — (PDF) [file pone.0168805.s004.pdf]

Supplementary Data

Supplementary S2 Table. Multi-Parametric Heat Map Analysis - Parameter values normalization table

Parameter	Units	-1*	0*	1**
**After Breakfast**	mg/dl	70	126	241.3
**After Dinner**	mg/dl	70	126	227.9
**After Lunch**	mg/dl	70	126	217.3
**AUC180/iAUC**	%	0	0	94.5
**Before Breakfast**	mg/dl	70	126	183.0
**Before Dinner**	mg/dl	70	126	192.9
**Before Lunch**	mg/dl	70	126	194.2
**BGRI**	A.U	1.1	2.5	14.2
**CONGA2**	mg/dl	10	10	35.7
**CONGA4**	mg/dl	10	10	43.7
**CONGA6**	mg/dl	10	10	47.3
**Cortisol**	ng/ml	50	230	268.8
**CV**	%	14.3	17.2	33.5
**FBG**	mg/dl	70	126	230.0
**HbA1c**	%	4	5.7	8.8
**MAGE**	mg/dl	10	30	113.7
**MAX Breakfast**	mg/dl	126	180	276.3
**MAX Dinner**	mg/dl	126	180	250.3
**MAX Lunch**	mg/dl	126	180	254.6
**Mean 24**	mg/dl	70	116	201.7
**Mean Morning**	mg/dl	70	126	185.8
**Mean Night**	mg/dl	70	126	203.3
**MGTT**	mg/dl h	10	25	104.0
**MODD**	mg/dl	10	10	55.2
**M-Value**	A.U	0.0	18.0	25.2
**SD 24**	mg/dl	10	20	44.5

* "0" and "-1" are the upper and lower levels of an estimated normal interval for each parameter

** "1" is the maximum value of each parameter recorded in this trial.
