# Supplementary material for: Effect of Peripheral Electrical Stimulation (PES) on Nocturnal Blood Glucose in Type 2 Diabetes: A Randomized Crossover Pilot Study
Source: PLoS One. 2016 Dec 20;11(12):e0168805. doi: 10.1371/journal.pone.0168805 (PMC5173375; doi:10.1371/journal.pone.0168805)
Supplement: S3 Table — (PDF) [file pone.0168805.s005.pdf]

Supplementary Data

Supplementary S3 Table. Experimental Data

**Control**		**Patients**										
Parameter	***Units***	***002***	***003***	***004***	***005***	***006***	***007***	***008***	***009***	***010***	***011***	***012***
Mean 24	mg/dl	111.38	134.44	107.89	128.70	201.66	201.29	137.21	134.52	131.33	156.08	150.84
Mean Morning	mg/dl	101.00	118.00	112.12	109.33	185.83	143.71	136.10	110.17	110.62	147.33	132.00
Mean Night	mg/dl	105.37	127.46	104.31	96.12	203.31	179.42	161.68	108.20	118.63	143.75	117.46
Mean Day	mg/dl	118.80	134.60	107.89	140.50	204.40	212.20	162.50	145.22	137.60	163.70	154.30
SD 24	mg/dl	21.11	37.48	20.51	32.23	40.49	44.11	44.51	37.08	32.40	42.11	38.41
Before Breakfast	mg/dl	112.60	119.40	131.50	118.00	178.00	178.50	132.67	120.70	128.70	145.00	133.70
Before Lunch	mg/dl	95.30	105.60	110.67	141.80	155.03	194.17	123.92	105.80	119.96	135.10	139.21
Before Dinner	mg/dl	98.67	127.20	98.70	131.33	165.80	192.92	156.11	111.92	127.75	134.90	122.08
After Breakfast	mg/dl	121.32	139.14	112.52	167.29	241.25	214.04	176.57	141.19	194.46	160.57	164.24
Afte Lunch	mg/dl	140.77	160.57	103.21	111.63	217.26	202.21	191.21	182.75	142.61	204.49	212.61
Afte Dinner	mg/dl	131.43	141.57	99.57	126.46	227.89	218.98	225.19	153.36	166.93	155.31	190.18
MAX. Breakfast	mg/dl	151.75	155.67	169.00	185.66	276.25	255.00	203.00	219.00	226.25	203.00	181.67
MAX. Lunch	mg/dl	152.40	179.00	132.75	164.80	240.20	234.00	204.00	199.00	168.00	231.20	253.70
MAX. Dinner	mg/dl	143.00	165.50	148.40	158.00	250.33	229.70	249.67	174.00	177.00	171.20	194.25
AUC180/iAUC	%	0.00	17.21	4.38	5.26	94.54	50.54	36.38	13.63	10.71	43.41	26.30
MGTT	mg/dl h	41.92	68.25	50.00	50.08	50.08	99.29	91.83	70.00	72.08	49.13	35.83
M-Value	A.U	4.23	9.98	4.30	6.70	23.07	25.20	8.59	10.00	8.90	13.80	12.50
%CV	%	18.95	27.88	19.01	25.04	20.08	21.91	32.44	27.56	24.67	26.98	25.46
BGRI	A.U	1.14	3.25	1.19	2.23	14.20	12.19	6.39	3.08	2.93	6.10	5.18
MODD	mg/dl	17.51	34.36	20.98	23.60	47.46	46.65	49.57	28.37	28.05	55.25	24.69
MAGE	mg/dl	54.78	62.32	40.68	65.18	90.54	80.08	113.74	108.17	78.16	79.45	80.81
COGNA6	mg/dl	17.34	35.28	19.15	31.18	38.45	39.24	47.33	28.94	30.03	32.52	33.13
COGNA4	mg/dl	20.66	30.14	18.25	27.56	37.92	35.18	43.66	37.27	30.63	36.72	31.13
COGNA2	mg/dl	18.28	19.10	21.07	21.73	28.22	27.75	31.91	32.38	27.60	30.02	28.81
INS	µIU/ml	2.95	2.70	3.47	3.55	7.77	8.30	8.86	3.09	3.36	3.48	3.05
Cortisol	ng/ml	213.40	243.60	156.00	169.20	94.80	268.80	103.20	160.80	128.80	130.60	194.10
FBG	mg/dl	113.00	84.00	138.00	130.00	230.00	207.00	156.00	146.00	123.00	172.00	167.00
HbA1c	%	8.10	7.10	6.60	6.50	8.80	7.40	6.90	7.20	6.30	7.60	7.60

PES		Patients										
Parameter	***Units***	***002***	***003***	***004***	***005***	***006***	***007***	***008***	***009***	***010***	***011***	***012***
Mean 24	mg/dl	102.25	125.26	112.25	121.51	190.83	157.38	123.08	127.67	121.27	147.86	126.27
Mean Morning	mg/dl	93.69	80.93	110.55	98.09	172.12	118.51	108.77	100.57	102.26	130.10	110.44
Mean Night	mg/dl	90.27	98.57	103.59	101.90	178.64	146.69	138.57	95.94	109.32	138.15	88.46
Mean Day	mg/dl	116.20	134.60	122.40	133.40	207.20	153.90	120.50	139.50	125.14	146.90	137.20
SD 24	mg/dl	18.30	39.46	17.61	26.56	36.95	43.88	30.02	42.75	27.28	25.04	29.59
Before Breakfast	mg/dl	100.80	88.25	124.30	100.54	183.00	138.20	103.56	117.89	113.50	132.56	113.71
Before Lunch	mg/dl	83.20	103.33	104.80	109.33	164.00	132.44	90.90	94.42	118.28	124.30	109.93
Before Dinner	mg/dl	95.80	117.67	97.00	130.03	149.56	144.38	103.70	99.67	118.54	128.11	110.50
After Breakfast	mg/dl	113.79	135.32	125.19	127.57	202.67	208.95	156.76	149.90	137.90	149.90	132.50
Afte Lunch	mg/dl	118.34	174.39	116.38	115.09	209.34	143.76	118.32	174.00	135.81	152.11	182.91
Afte Dinner	mg/dl	113.20	127.81	129.93	141.43	214.48	216.25	134.05	133.81	128.61	164.00	143.74
MAX. Breakfast	mg/dl	141.75	165.00	149.00	166.75	240.33	222.67	200.33	221.33	198.00	175.00	158.00
MAX. Lunch	mg/dl	128.60	203.25	144.33	151.20	254.60	186.00	132.70	207.00	149.00	178.50	206.40
MAX. Dinner	mg/dl	123.00	163.33	153.75	186.20	250.33	254.00	157.30	153.67	167.25	185.00	156.00
AUC180/iAUC	%	0.00	3.46	0.00	2.09	53.23	29.76	4.12	15.95	5.56	7.59	9.77
MGTT	mg/dl h	36.38	52.46	50.88	60.29	52.96	103.96	77.96	54.67	51.17	49.00	42.67
M-Value	A.U	5.50	10.32	3.90	5.40	19.64	15.70	7.40	11.28	6.50	6.90	9.00
%CV	%	17.90	31.50	15.69	21.86	19.36	27.88	24.39	33.49	22.50	16.93	23.44
BGRI	A.U	1.44	3.65	0.78	1.73	10.51	5.20	2.11	3.38	1.54	3.26	2.04
MODD	mg/dl	18.10	29.88	19.42	23.89	48.85	36.98	25.66	27.10	22.03	22.44	22.06
MAGE	mg/dl	40.46	75.96	36.63	49.80	84.49	75.79	60.08	89.93	66.78	48.27	66.15
COGNA6	mg/dl	16.53	33.48	14.20	24.84	29.20	43.27	26.80	39.49	26.59	19.22	24.12
COGNA4	mg/dl	18.87	31.84	15.12	22.80	32.15	38.92	28.80	41.99	28.44	20.78	24.51
COGNA2	mg/dl	16.55	22.74	13.44	20.61	26.83	30.37	22.91	35.74	27.01	19.33	23.65
INS	µIU/ml	3.83	2.75	3.58	3.28	6.11	4.16	8.83	2.78	1.06	5.03	3.47
Cortisol	ng/ml	78.80	176.00	113.80	69.80	57.80	133.20	107.00	169.20	71.00	128.20	116.40
FBG	mg/dl	104.00	75.00	135.00	114.00	182.00	155.00	91.00	125.00	107.00	157.00	133.00
HbA1c	%	6.60	6.70	6.60	5.90	8.20	7.00	6.70	6.70	5.80	7.60	6.90
